# Can Machines Identify Pain Effects? A Machine Learning Proof of Concept to Identify EMG Pain Signature

**DOI:** 10.3390/bioengineering13020141

**Published:** 2026-01-26

**Authors:** Klaus Becker, Franciele Parolini, Venicius de Paula Silva, João Paulo Vilas-Boas, Thomas Graven-Nielsen, Ulysses Ervilha, Márcio Goethel

**Affiliations:** 1 Porto Biomechanics Laboratory, University of Porto, 4200-450 Porto, Portugal; fcsp@ess.ipp.pt (F.P.); jpvb@fade.up.pt (J.P.V.-B.); gbiomech@fade.up.pt (M.G.); 2 Center of Research, Education, Innovation and Intervention in Sport, Faculty of Sport, University of Porto, 4200-450 Porto, Portugal; ulyervil@usp.br; 3 Center for Rehabilitation Research (CIR), ESS, Polytechnic of Porto, 4200-072 Porto, Portugal; 4 Laboratory of Physical Activity Sciences, School of Arts, Sciences, and Humanities, University of São Paulo, São Paulo 03828-000, Brazil; venicius@usp.br; 5 Center for Neuroplasticity and Pain (CNAP), Department of Health Science and Technology, Faculty of Medicine, Aalborg University, 9260 Aalborg, Denmark; tgn@hst.aau.dk

**Keywords:** experimental pain, pain theory, artificial intelligence, XGBoost

## Abstract

This study introduces a machine-learning-based approach for identifying “pain signatures” using electromyography data from volunteers undergoing acute pain. Leveraging the XGBoost algorithm, our method analyzes electromyography features (variance, mean absolute deviation, integral, peak, and entropy) to classify muscle contractions as painful or non-painful. Fifteen participants performed controlled elbow flexion tasks under three conditions: during painful and painless conditions. The results revealed that electromyographic peak and integral activity were key predictors of pain states, with the model achieving 73% sensitivity in distinguishing painful from painless conditions. Interestingly, placebo-induced responses with less intense pain exhibited muscular adaptations similar to, but less extensive than, those observed under actual pain. These findings underscore the potential of machine learning to enhance pain assessment by providing a non-verbal, objective method for analyzing neuromuscular adaptations, paving the way for personalized pain management and more accurate monitoring of musculoskeletal health.

## 1. Introduction

Pain, a complex sensory and perceptual experience, is intimately linked to alterations in motor control, resulting in changes to muscle command and coordination [1]. These changes, often manifested as protective motor strategies driven by spinal mechanisms [2], may become maladaptive over time and ultimately hinder neuroplasticity [3]. Currently, the theory states that it is not exactly a regular decrease or increase in motor command, but rather that there is a difference, at various levels, such as changes in the recruitment strategy of synergistic muscles [4], with different activation within muscles depending on the task [5]. It is well known that muscle activation strategies adapt to mechanical constraints [6], muscle fatigue [7], and acute pain [8,9,10].

There is an open hypothesis in the literature claiming that the subject’s muscle coordination strategies can make some people more at risk of developing skeletal muscle disorders due to greater mechanical stress on muscles and joints [11]. Some authors argue that the large intersubject variability that exists in variables related to muscle activity may prevent changes caused by pain from being easily identified, even during the same task [8], due to their idiosyncrasy [12]. This variability challenges the idea of a “universal” motor response to pain and highlights the complexity of neuromuscular adaptations to painful stimuli. Understanding these patterns would be important to understand more broadly the behavioral effects that pain can particularly have on a person. Furthermore, the placebo effect is elicited by elements beyond the active intervention itself [13] and may rely on neurotransmitter release related to expectancy and conditioning [14]. It becomes essential to account for these effects when investigating the physiological mechanisms of pain modulation.

The use of machine learning allows for, in addition to identifying the interaction between numerous variables, the identification of the weighting of the influence of parameters such as time, intensity, complexity, and dispersion of electromyographic (EMG) data in detecting the presence of pain [15]. A tool capable of detecting the presence of pain by analyzing the interrelation of EMG variables would be of great value as an objective way of assessing pain’s possible consequences on movement control. Furthermore, this type of EMG data processing potentially allows for the identification of patterns that are sufficiently robust to be interpreted as an “EMG pain signature”.

The present study proposes to develop a machine-learning-based tool capable of identifying the “EMG pain signature” through the analysis of EMG signals. The tool utilizes XGBoost, a scalable algorithm that combines the outputs of multiple decision trees while minimizing overfitting and underfitting through regularization [16]. Using EMG features such as variance (VAR), mean absolute deviation (MAD), integral (INT), EMG peak, and approximate entropy (AppEn) of the EMG signal, the tool analyses muscle contractions to classify them as painful or non-painful.

The objective of this study is, based on machine learning techniques, to identify and characterize muscle activity patterns associated with pain. Specifically, the study aims to (1) detect a common EMG pain signature among all subjects by analyzing before and during pain induced by an intramuscular injection of hypertonic saline, and (2) to identify the non-painful EMG signature, where the muscular responses were obtained immediately after the intramuscular injection of isotonic solution inducing minimal pain (placebo).

## 2. Materials and Methods

### 2.1. Subjects

This is a secondary analysis of previously published data, and details of the entire experimental setup can be accessed elsewhere [17]. Fifteen subjects (eleven men, four women) who were 26 ± 5 years, 1.75 ± 0.09 m tall, and had 70 ± 11 kg of body mass participated in the experiment. The sample size of 15 participants was determined by the availability of data from the original experimental study; however, the sample size is consistent with similar studies using physiological data for machine-learning-based studies. All subjects did not suffer from any musculoskeletal condition prior to the experiment and had no musculoskeletal pain in the last 24 h or continued pain in the last 3 months. Volunteers received information about the experiment, and subsequently, written consent was obtained prior to inclusion. The study was approved by the local Ethics Committee (VN2003/61) and conducted according to the Helsinki Declaration.

### 2.2. Apparatus and Movement

**Figure 1:**
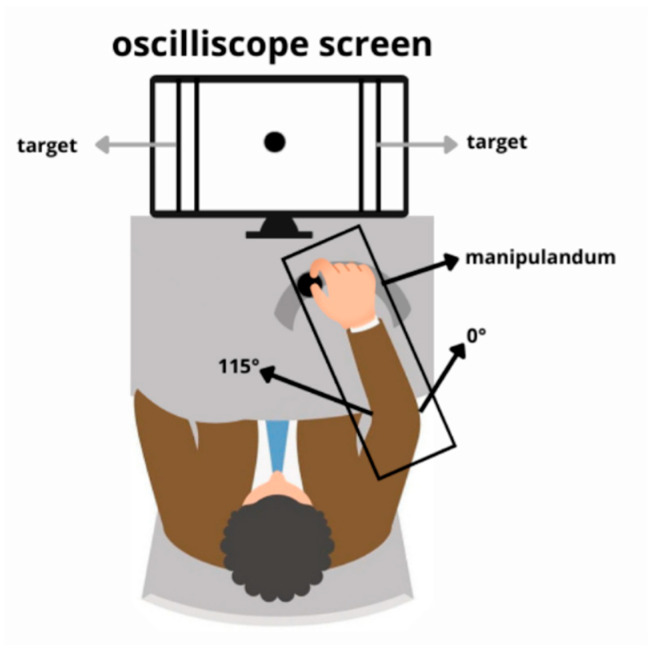
Experimental setup: An oscilloscope displayed real-time elbow movement data captured by an electrogoniometer positioned directly in the subject’s view. The target angles were established at 115° and 45°. Adopted from previous work [17].

With the arm and forearm supported on a manipulandum, elbow flexion and extension movements were performed uninterruptedly for two minutes, with the aim of hitting targets placed at the end of the permitted range of motion. Figure 1 illustrates the experimental setup, a detailed description of which is available in a previously published study [17].

### 2.3. Protocol

This study constitutes a secondary analysis of data obtained from a controlled, within-subjects, crossover experimental design. The original data collection was conducted at the Laboratory for Experimental Pain Research, Center for Sensory-Motor Interaction, Aalborg University, Fredrik Bajers Vej 7D-3, 9220, Aalborg, Denmark. The subsequent secondary data analysis (machine learning classification) was performed at LABIOMEP from University of Porto, Portugal. Two sessions were conducted one week apart. All contractions of the elbow flexion identified during the two minutes of the task were considered. In each session, volunteers performed the task under two conditions: (1) before the onset of experimental muscle pain and (2) immediately after hypertonic saline solution injection or (3) after isotonic saline injection. The orders of conditions 2 and 3 were randomized between sessions.

Hypertonic or isotonic saline was injected into the biceps brachii muscle to induce pain. Elbow flexion movements were assessed in two epochs: before saline infusion and immediately after infusion (hypertonic or isotonic), respectively, indicating pain and painless/placebo. EMG was recorded from the biceps brachii muscle to analyze muscle activity during pain/placebo. EMG intensity was normalized by the respective peak of maximal voluntary isometric contraction (MVIC) recorded prior to the experimental pain induction to allow for comparison across subjects and conditions.

### 2.4. Experimental Muscle Pain

One bolus of 1.5 mL of sterile hypertonic saline (5.8%) solution was injected intramuscularly into the biceps muscle (lateral head) at a rate of 180 mL/h via a disposable stainless needle (27 g, 40 mm) connected via a tube (IVAC, G30303) to an infusion pump (ALARIS medical systems, Asena, UK). A 10 cm electronic visual analogue scale (VAS), where 0 cm indicated “no pain” and 10 cm “intolerable pain”, was used to score the pain intensity. The signal from the VAS was recorded continuously. The subjects were allowed to adjust the values using the hand not involved in the exercise, and they were asked to focus on the VAS in the breaks between the individual trials. The mean VAS scores obtained during and between the trials were calculated.

### 2.5. Data Analysis

The angular position was digitally filtered (low-pass, fourth-order, and zero-phase-lag Butterworth filter with a 10 Hz cut-off frequency) and differentiated to obtain velocity and acceleration. EMG signals were digitally band-pass-filtered from 20 to 400 Hz (Butterworth) and low-pass-filtered (Butterworth) with a 50 Hz cut-off frequency, and EMG peak and INT data were normalized by the maximal voluntary isometric contraction recorded before the dynamic contractions. Extracted parameters for this model were VAR, MAD, INT, and peak, where calculation from individual trials was performed in accordance with Vonsevych et al. [18] and AppEn in accordance with Chen et al. [19]. INT was calculated over the movement epoch (defined from the onset of biceps brachii activity to the offset of acceleration). To eliminate the confounding influence of movement duration on the integral value, INT was time-normalized by dividing the raw integral by the epoch duration and multiplying by 100. This normalization ensured that INT reflects the average magnitude of muscle recruitment independent of the temporal variations in the task.

### 2.6. Machine Learning Analysis

The experimental variables described above (VAR, MAD, INT, peak, and AppEn) served as input features for an XGBoost (eXtreme Gradient Boosting) model. XGBoost is a powerful machine learning algorithm that utilizes an ensemble of decision trees to make predictions. XGBoost is a leading algorithm for structured, tabular data, akin to a spreadsheet or database table that includes built-in techniques to prevent overfitting, a common issue in machine learning where models learn the training data too well and fail to generalize to new, unseen data [20]. In the present analyses, the model was trained using 70% of all data before and during painful contractions (1654 contractions) and tested on 30% of the data before and during painful contractions (710 contractions). Initial training with pain data allowed the model to learn to associate EMG signal characteristics with the presence of pain or no pain. Furthermore, when applying the algorithm to contractions performed during placebo conditions, since theoretically there is no nociceptive signal, we used only the post-placebo injection epoch (1235 contractions). SHAP (Shapley Additive Explanations) values were used to explain the contribution of each variable to the model’s accuracy. To enhance the explainability and interpretability of deep machine learning models, SHAP values offer a valuable visualization tool that translates the model’s output into terms understandable by human intuition [20,21].

### 2.7. Statistical Analysis

The normality of data distribution was verified with the Shapiro–Wilk test. Results are presented by mean and standard deviation. To compare the mean EMG variables between contractions classified as painful and non-painful, Student’s independent *t*-test was employed. Additionally, paired *t*-tests were applied to assess differences between pre- and post-injection contractions. Cohen’s *d* criteria were used to calculate the power of analyses (>0.2: small; >0.50: moderate; >0.80: large) [22]. All tests were performed using SPSS Statistics version 27 (IBM Corporation, Armonk, NY, USA), and significance was set at *p* < 0.05.

## 3. Results

**Figure 2:**
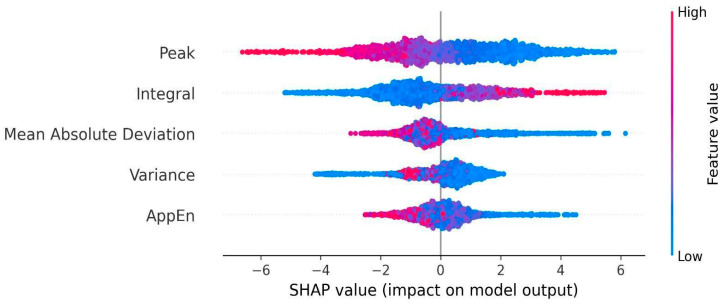
Beeswarm plot of SHAP values, where each dot represents a single contraction. The variables are ranked by its predictive power for the model from top to bottom. The color bar indicates the direction of the relationship of the variable for the prediction of the model, for example, the higher the peak EMG values, the less pain the subject reported. These results were obtained from the 70% of the data that was used in the training set.

**Figure 3:**
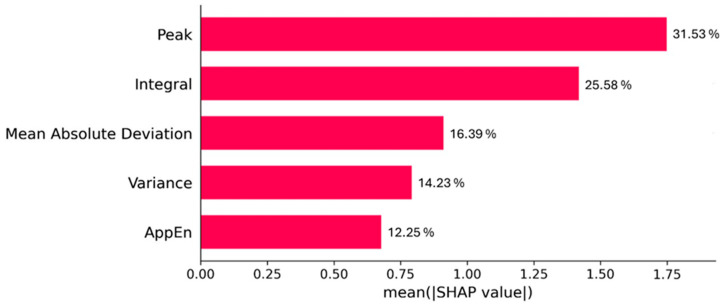
Hierarchical average global impact on EMG variable model expressed by SHAP (Shapley Additive Explanations) values, obtained from the 70% of the data that was used in the test.

The analysis of test performance revealed that EMG peak and INT had opposite effects on the model: lower peaks and higher INT increased the probability of the model classifying the contraction as painful. Figure 2 shows the directionality of the SHAP values. Also, these were the most important predictors for differentiating between pain and no-pain states in the training set. Figure 3 shows the weight of the variable obtained from the XGBoost model. Consequently, a high peak in the EMG signal was associated with the absence of pain, while increases in INT were correlated with the presence of pain.

**Figure 4:**
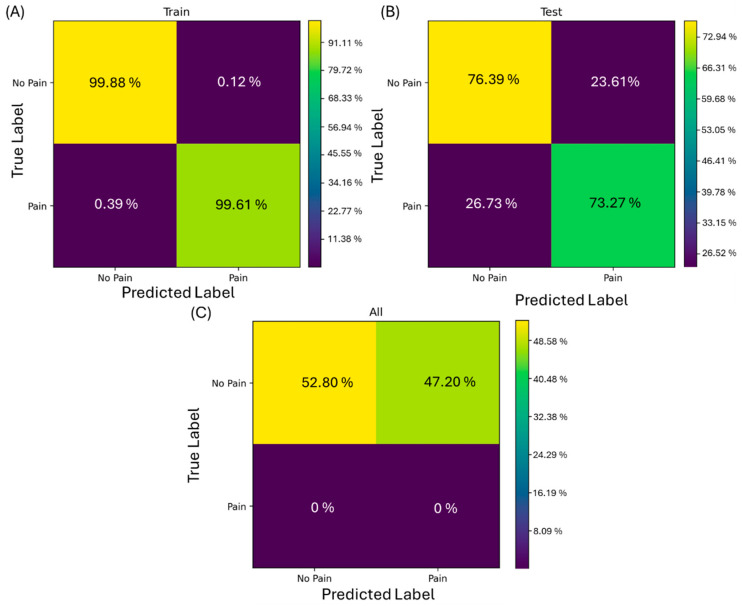
Confusion matrix with contractions evaluated. On the *y* axis, the true label as a percentage of contractions of all subjects performed during pain and no pain is shown; on the *x* axis, the predicted label when the model recognizes contractions with pain patterns and/or non-painful patterns is shown. (**A**) Confusion matrix of training data prediction of number of contractions with painful features and non-painful features. (**B**) Confusion matrix of test data prediction. (**C**) Confusion matrix of contractions after isotonic injection. Contractions count: (**A**) training set: 1654 contractions; (**B**) testing set: 710 contractions; (**C**) placebo: 1235 contractions.

Figure 4 presents the confusion matrices for different stages of the machine learning model’s training and testing (i.e., sensitivity and specificity). In Figure 4A, the results of the training set demonstrate high accuracy in classifying contractions as painful (true positives) and painless (true negatives). Figure 4B illustrates the model’s performance on the testing set (before and after hypertonic injection EMG recordings), with a slight reduction in accuracy compared to the training set. After training, the model was tested, with results blinded to the model, during pre- and post-administration of hypertonic saline (inducing pain), as shown in Figure 4B. The result of 73.27% indicates that the algorithm was able to identify EMG patterns associated with pain in the data and was able to recognize similar patterns under both conditions. The model was also tested with EMG placebo data. Considering that the placebo does not induce pain or it induces a very low-intensity pain, only the post-injection moment was analyzed. The accuracy of 52.8% suggests that the placebo effect may generate muscle responses like those observed under actual pain conditions in approx. half of the non-painful contractions.

To evaluate the model’s ability to generalize the identified “EMG pain signature” and ensure that its performance was not inflated by subject-specific baseline characteristics, independent *t*-tests were performed to compare the training and testing datasets. For the baseline (non-painful) condition, significant differences were observed between the training and testing sets in key predictive features, specifically EMG peak and INT. Similar statistical discrepancies were found during the painful condition, with significant differences between datasets in VAR, MAD, INT, and EMG peak, see Table 1.

Analysis of the EMG parameters following the placebo injection, comparing trials subsequently classified as painful and non-painful by the model, revealed statistically significant differences (Table 2). Paired *t*-tests indicated that the EMG peak and INT parameters were significantly different between the two classified groups.

**Figure 5:**
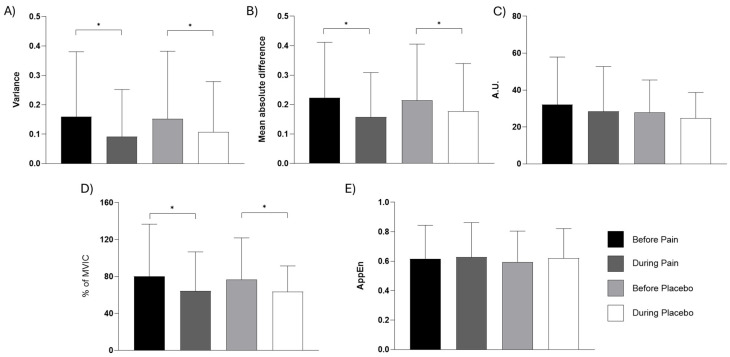
Mean and standard deviation of EMG variables during pain condition and immediately after placebo injection. (**A**) Variance (VAR); (**B**) mean absolute difference (MAD); (**C**) integral (arbitrary units); (**D**) peak EMG (% of maximum voluntary isometric contraction); (**E**) AppEn (approximate entropy). * *p* < 0.05.

Figure 5 presents the means and standard deviations of the analyzed variables (VAR, MAD, INT, peak, and AppEn) before and after pain induction or placebo. Statistical analysis using Student’s *t*-test revealed significant differences in EMG variables during experimental pain (Figure 5). Specifically, there was a decrease in variability (VAR, *p* < 0.001, d = 0.348), MAD (*p* < 0.001, d = 0.378), and peak (*p* < 0.001, d = 0.311). Similarly, under control conditions with isotonic solution, a decrease was observed in the same variables: VAR (*p* < 0.001, d = 0.532), MAD (*p* < 0.001, d = 0.463), and peak (*p* < 0.001, d = 0.553).

## 4. Discussion

### 4.1. General Discussion

By employing machine learning tools, researchers can investigate the effects of pain from a broader perspective, including obtaining the respective weights and influences of the variables that jointly compose the behavioral outcome. This tool was crucial to our study, enabling us to explore complex patterns and interactions in pain-related variables that would be difficult to uncover using traditional methods. We successfully achieved our first aim, demonstrating that the predictive model could accurately distinguish between painful and non-painful contractions using EMG variables and consistently detect the “EMG pain signature” across subjects. However, the second aim that the model would identify the non-painful muscular responses compared to actual pain was rejected. Instead, the results showed that placebo-induced responses share similarities with pain-induced responses; in both conditions, the model identified a concomitant reduction in EMG peak, VAR, and MAD. This suggests that the placebo effect elicits neural and muscular adaptations resembling those of actual pain.

This study demonstrates the potential of XGBoost as a powerful tool in identifying distinct muscular activity patterns associated with pain and low-pain/pain-free conditions. This result may indicate that pain induces a reconfiguration of muscle activity that can be detected through specific EMG variables. Furthermore, the present model could capture these changes with high sensitivity (73. 3%, Figure 4B). A particularly innovative aspect of this study was that a machine learning model based on EMG variables can differentiate between painful and non-painful contractions (Figure 4A,B), suggesting a potential specific EMG pain signature, thereby supporting our first aim. Although both scenarios exhibited similar muscular patterns, indicative of comparable physiological responses, the model was able to detect subtle differences. For example, higher values of INT are important for the model to classify the condition as “painful”. However, inferential statistics do not identify these subtle changes that are so important for classification. These findings suggest that the expectation of pain associated with the placebo effect might trigger muscular responses like those observed in actual pain conditions. Similar studies [23,24] have demonstrated the efficacy of XGBoost in analyzing EMG signals to identify patterns of muscular dysfunction. The combination of variables such as entropy and variability in EMG signals has been particularly useful in differentiating between signals of painful and placebo activity. Furthermore, the evidence that pain alters the coordination and recruitment of motor units, impacting the variability in the EMG signal, reinforces the notion that a possible “pain signature” can be identified through specific patterns in EMG variables [18,25].

These findings suggest that the expectation of pain associated with the placebo effect might trigger muscular responses comparable to those observed in actual pain conditions. While previous research has utilized machine learning to detect muscular patterns associated with injury or dysfunction, our findings extend this by identifying a specific EMG pain signature that appears even during placebo-induced conditions. This highlights that measuring pain is far beyond asking them to rate the pain, because pain anxiety could evoke muscle responses already. Also, an objective classification of pain through EMG must account for central mechanisms related to the “whole experience of pain”, a factor often overlooked in binary ‘pain vs. no-pain’ classification models.

### 4.2. Mechanism Insights

The presence of similar EMG patterns in both painful and part of placebo conditions suggests that the perception of pain can evoke protective muscular responses, mediated by central mechanisms and expressed at the periphery [26]. This response can be seen as part of an evolutionary adaptation mechanism, where the motor system adjusts to minimize potential additional damage. When the body perceives a threat (real or potential), it may adopt motor strategies that increase stiffness and decrease muscle variability to protect the affected area. This includes changes such as a decrease in variability and decreases in entropy, reflecting the body’s attempt to stabilize the affected region [27]. However, the algorithm was also able to differentiate between pain and placebo based on small variations in the intensity of these muscular responses. For example, while entropy decreased in both conditions, the reduction was more pronounced in pain, indicating a higher level of muscular reorganization. This suggests that, despite similar responses, the intensity and coordination of motor responses may be more pronounced in the presence of pain.

Neurophysiological studies indicate that the placebo effect is not merely a psychological phenomenon but involves actual changes in neural and muscular activity [28]. The activation of brain circuits related to pain modulation, such as the prefrontal cortex, nucleus accumbens, and periaqueductal gray matter, may induce alterations in muscular response. While the precise mechanisms are not fully understood, it is plausible that these circuits, through various pathways influencing motor cortex activity and spinal motor neuron excitability, contribute to changes in EMG variables like signal entropy and variability [29,30], as well in placebo [28]. The reduction in entropy observed during pain suggests a simplification of the muscular activation pattern, coordinated by the central nervous system to reduce movement and prevent additional damage [31], which may be associated with a state of hypervigilance or adaptation to acute pain [32,33]. However, this could be deleterious in the long term, since a loss in diversity could be harmful to certain structures. This adjustment can be seen as an attempt by the body to “compartmentalize” movement, recruiting more stable but less flexible motor patterns to minimize the risk of exacerbating pain. Our results show that AppEn, despite been relevant, carries a weight of 12% to allow the model to distinguish between the pain and no-pain conditions.

Although the placebo effect can induce similar physiological responses to actual pain, the intensity of these responses tends to be lower. This is well explained by the absence of a real noxious stimulus [34]. During the placebo condition, the expectation of pain can activate neural networks associated with pain but without the same depth of muscular reorganization necessary to cope with a real painful stimulus [28].

The findings of EMG pain signatures during the placebo condition are a very impactful result. The model separated painful and non-painful contractions mainly based on the two most important variables, peak and INT, suggesting that the placebo condition elicits a similar signature on the EMG pattern to painful contractions. Placebos are inert substances or sham interventions commonly used in scientific studies to understand the effects of a given intervention beyond subjects’ belief in the treatment’s efficacy. They help isolate the true impact of the intervention by comparing it to an inert substance or treatment [35]. In the current experimental pain induction model, in fact, it can be classified as a nocebo effect, since it had the same effect on muscle activation as the painful condition [36]. Our algorithm found the same pattern in almost half of the contractions from the sample. Future studies should consider this bias presented in the experimental acute pain model. Also, future investigation should focus on knowing in more depth how factors such as expectation, belief, and conditioning can influence pain response in order to strengthen the model for research purposes.

## 5. Limitations and Future Work

A limitation relates to the experimental pain model employed, as since pain is induced in a highly controlled environment, this can conditionate the signature. Therefore, the ‘EMG pain signature’ identified by our classifier may be specific to acute, experimentally induced pain and may not be fully replicated in clinical pain, but this works as a proof of concept that the EMG signal contains a pattern recognizable for an algorithm. Future work should further investigate this possible difference in chronic pain conditions. Another limitation of this study is the sample size, which was predetermined by the availability of data from the original experimental protocol. While the within-subjects design inherently controls inter-subject confounders, the small sample may not fully capture the extensive inter-individual variability inherent in pain perception and EMG responses. Consequently, the generalizability of the XGBoost model trained to a broader, more heterogeneous population might present some differences. In clinical chronic pain populations, neuromuscular adaptations are often influenced by long-term neuroplastic changes and central sensitization, which may result in different or more complex EMG patterns than those observed in this controlled environment [37,38]. Consequently, this study should be viewed as a proof of concept, and the identified signature serves as a hypothesis for future validation in clinical settings.

The successful identification of a potential “EMG pain signature” using the XGBoost model represents a significant step toward developing an objective diagnostic tool. This approach holds particular promise for managing musculoskeletal and tendinous pain (which constitutes the majority of cases presenting in the healthcare system [39]). Given that current imaging techniques (such as X-ray or magnetic resonance imaging) are often non-specific for the underlying functional pain in these conditions, our machine learning model offers a non-invasive, cost-effective alternative diagnostic pathway. By quantifying the specific neuromuscular adaptations associated with pain, this tool could serve to directly diagnose a pain state rather than relying on structural abnormalities that may or may not correlate with the patient’s symptoms. This objective, functional assessment has the potential to guide clinical decision-making more efficiently than current nonspecific methods, paving the way for targeted, personalized interventions from the initial healthcare consultation.

While the current results regarding a potential ‘EMG pain signature’ are specific to acute experimental pain and remain speculative for clinical or neuropathic conditions, they provide a vital proof of concept. This study establishes a methodological basis for future research to explore these patterns in larger, more heterogeneous clinical cohorts, where chronic pain mechanisms may further alter neuromuscular signatures.

## 6. Conclusions

In the current study, it was possible to recognize patterns suggestive of an “EMG pain signature” using machine-learning-based tools, specifically the XGBoost algorithm, applied to EMG signals. The analysis of EMG variables revealed patterns that can be associated with the presence of pain as well as with placebo effects caused by intramuscular isotonic infusions. Ultimately, this work contributes to the broader goal of improving pain assessment methods by offering a non-verbal, objective approach to understanding how pain affects motor function. In achieving this, it lays the groundwork for future applications in personalized pain management and musculoskeletal health monitoring.

## Figures and Tables

**Table 1:** Results of the paired student *t*-tests for EMG parameters between contractions classified as painful and non-painful by the algorithm.

Contraction Variables	Classified as Non-Painful	Classified as Painful
PEAK	0.385	<0.001
INT	0.269	<0.001
MAD	<0.001	<0.001
VAR	0.023	<0.001
APPEN	0.795	0.619

**Table 2:** Results of the paired student *t*-tests for EMG parameters after placebo injections. The table presents the values from paired *t*-tests comparing five distinct EMG parameters between two trial groups: those classified by the XGBoost algorithm as painful and those classified as non-painful following the placebo administration. A statistically significant difference was observed for the peak and INT parameters.

PEAK	0.038
INT	0.040
MAD	0.814
VAR	0.623
APPEN	0.525

## Data Availability

The data presented in this study are openly available in the figshare repository. Doi: http://doi.org/10.6084/m9.figshare.25723794.

## Abbreviations

The following abbreviations are used in this manuscript:

EMG	Electromyography
XGBoost	eXtreme Gradient Boosting
VAR	Variance
MAD	Mean Absolute Deviation
INT	Integral
AppEn	Approximate Entropy
MVIC	Maximal Voluntary Isometric Contraction
VAS	Visual Analogue Scale
SHAP	Shapley Additive Explanations
